# Male involvement in family planning use and its determinants in Ethiopia: a systematic review and meta-analysis protocol

**DOI:** 10.1186/s13643-022-01891-x

**Published:** 2022-02-01

**Authors:** Etsay Woldu Anbesu, Setognal Birara Aychiluhm, Zinabu Hadush Kahsay

**Affiliations:** 1 Department of Public Health, College of Medical and Health Sciences, Samara University, Samara, Ethiopia; 2 School of Public Health, College of Health Science, Mekelle University, Mekelle, Tigray Ethiopia

**Keywords:** Pooled prevalence, Determinants, Male involvement, Family planning use, Ethiopia

## Abstract

**Background:**

The need to include males who require joint spousal decisions is critical in achieving key reproductive health indicators. Low involvement of males in family planning use is one of the contributing factors for low contraceptive use in Ethiopia. Despite this, there are inconsistent findings on the prevalence and determinants of male involvement in family planning use in Ethiopia. Thus, this systematic review and meta-analysis aimed to determine the pooled prevalence of male involvement in family planning use and its determinants in Ethiopia.

**Methods:**

The Preferred Reporting Items for Systematic Reviews and Meta-Analyses guidelines will be used to develop the protocol. The online databases PubMed, CINAHL, Google Scholar, and unpublished gray literature will be searched to retrieve available articles from April 10 to August 11, 2021. The two authors will conduct selection of studies, data extraction, and quality assessment. The quality of the studies will be assessed using the Joanna Briggs Institute checklist. The chi-squared test and *I*-squared statistic will be used to examine heterogeneity among studies. Sources of heterogeneity will be investigated using subgroup analysis and meta-regression based on regions and residence (urban and rural). Publication bias will be examined by observation using funnel plots and statistically by Begg’s and Egger’s tests. A random-effects model will be used to estimate the pooled prevalence and its determinants of male involvement in family planning use.

**Discussion:**

The role of males in family planning and participation in contraceptive use improves women’s uptake and continuity of family planning use. Although there are studies on male involvement in family planning use, there are no synthesis research findings on the pooled prevalence of male involvement in family planning use and its determinants in Ethiopia. Therefore, the findings from this systematic review and meta-analysis will help the national health sector transformational plane emphasize the pooled prevalence and its determinants that drive low male involvement in family planning use in Ethiopia.

**Supplementary Information:**

The online version contains supplementary material available at 10.1186/s13643-022-01891-x.

## Introduction

“Male involvement in family planning refers to all organizational activities aimed at men as a discrete group which has the objective of increasing the acceptability and prevalence of the family-planning (FP) practice of either sex” [1]. Family planning is an effort by a couple to limit or space the number of children they have by using contraceptive methods [2]. Family planning use reduces unwanted pregnancy, induces abortion, and promotes birth spacing. Moreover, it also helps to reduce neonatal, infant, child, and maternal mortality [2, 3].

The need to include men who require joint spousal decisions is critical in achieving key reproductive health indicators [4–10]. However, male roles in couples’ fertility decision-making have been given less emphasis. There was a paradigm shift in male involvement and concerns from increasing contraceptive use and attaining demographic goals to gender equality and achieving various reproductive duties since the 1994 International Conference on Population and Development (ICPD) and the 1995 United Nations World Conference on Women [1, 11].

Globally, contraceptive method use varies from 69% in southeast Asia to 11% in Africa [12]. A qualitative review in sub-Saharan African countries showed low involvement of men in family planning use [13]. To achieve sustainable development goals (SDGs), the participation of men in reproductive health issues is crucial. Moreover, regulating fertility to the level of substitution is essential to increase economic development [14]. Family planning use can avert 32% and 10% of maternal and child mortality, respectively [15, 16].

Studies have shown that factors that contribute to low family planning coverage include the desire to have more children, lack of knowledge, lack of husband education, and negative perception toward family planning use, sex preference, religious prohibition, and low involvement of men [9, 13, 17, 18].

In Ethiopia, the decision on household-related issues, including fertility, mainly belongs to the husband. The low involvement of men in family planning use is one of the contributing factors for low contraceptive use and high unmet need in Ethiopia. Studies performed in different regions of Ethiopia showed the role of men in family planning use, and male participation in contraceptive use improves women’s uptake of family planning services [2, 4, 19–21]. The contraceptive use was low (41.4%), and there was a high unmet need for family planning (22%), which contributed to a high total fertility rate (TFR) of 4.6, maternal mortality of 412 per 100,000 live births, neonatal mortality of 30, infant mortality of 43, and underfive mortality rate per 1000 live births [2, 22].

Although the Ethiopian government set a target for a contraceptive prevalence rate of 55% by 2020 to achieve SDGs [23] and develop the National Guideline for Family Planning Services [24], low emphasis has been given to the role of men’s involvement. Dissipating this, there is a lack of nationally representative data on male involvement in family planning use in Ethiopia [2, 22]. Several studies have been conducted in different parts of the country on male involvement in reproductive health and utilization of family planning [4, 6, 25–27]. However, there are inconsistent findings on prevalence and its determinants of male involvement in family planning use [16–21, 25]. Therefore, this systematic review and meta-analysis protocol aimed to determine the pooled prevalence of male involvement in family planning use and its determinants in Ethiopia.

### Research question

- ❖ What is the pooled prevalence of male involvement in family planning use in Ethiopia?
- ❖ What are the determinants of male involvement in family planning use in Ethiopia?

### Objectives

- ❖ To determine the pooled prevalence of male involvement in family planning use in Ethiopia
- ❖ To identify determinants of male involvement in family planning use in Ethiopia

## Methods

### Study protocol and reporting

A systematic review and meta-analysis protocol will be prepared using the Preferred Reporting Items for Systematic Review and Meta-analyses (PRISMA) guidelines [28]. The PRISMA-P 2015 checklist will be used to report the protocol [29] (Additional file 1).

### Eligibility criteria

All observational studies, including cross-sectional, case–control, and cohort studies, will be included. Case reports, case series, conference reports, and expert opinions will be excluded from the review. Studies that reported the prevalence of male involvement in family planning use and its determinants among couples in Ethiopia will be included. Moreover, studies that reported only the prevalence of male involvement in family planning use or at least the measured association between determinant variables on male involvement in family planning use will be included. Studies that only investigate the qualitative approach of male involvement in family planning use will be excluded. If studies address both quantitative and qualitative findings, we will only consider the quantitative findings. Both community- and institution-based studies will be included. Studies published in the English language alone will be included. There will be a restriction on the date of publication since 1994, as this was the period for a paradigm shift in male involvement and concern from increasing contraceptive use and attaining demographic goals to gender equality and achieving various reproductive duties [11].

### PECO search guide

#### Population: Married men

Exposure: Determinates of male involvement in family planning use. Determinates are exposures that increase or decrease the likelihood of male involvement in family planning use in Ethiopia. The determinates can be marital status, the number of children, discussion with partner, knowledge on contraceptive use, ever used family planning methods, participation in community networks, etc.

Comparison: it is the reported reference group for each determinate in each study: marital status versus single, available children or not, good knowledge versus poor knowledge on contraceptives use, discussion of partners on family planning use or not, etc.

Outcome: The primary outcome of the study will be the pooled prevalence of male involvement in family planning use among married men in Ethiopia. The secondary outcome of the study will be determinates of male involvement in family planning use among married men in Ethiopia. Male involvement in family planning refers to the involvement of males in at least one of the following activities: discussion or spousal communication, support, approval, and contraceptive use of the husband.

### Searching strategy and study selection

Online databases including PubMed, Google Scholar, CINAHL, and unpublished gray literature will be used to search articles from April 10 to August 11, 2021. In addition, cross-reference searching of the included studies will be performed to include related studies. Removal of duplicates and irrelevant studies and inclusion of eligible studies will be performed. The two authors (EW and SB) will independently screen the studies. Studies that mentioned the objective of male involvement in family planning use with full text will be further evaluated for quality. The articles will be retrieved and exported to Endnote version 8 reference manager software to collect and organize search outcomes [30]. The search strategy procedure is shown in the PRISMA diagram (Additional file 2).

The search Medical Subject Heading (Mesh) terms will be developed using the authors’ keywords articles and PMID of sample index manuscripts on male involvement in family planning use, titles, and abstracts of studies. Then, search strategies will be developed using different Boolean operators, and modifications will be made based on the types of databases (Additional file 3).

### Quality assessments

Assessment of articles using their title, abstract, and full review of the manuscripts will be performed before the inclusion of articles in the final meta-analysis. The qualities of each article will be assessed by using the Joanna Briggs Institute Meta-Analysis of Statistics Assessment and Review Instrument (JBI-MAStARI) [31]. Particular attention will be given to a clear statement of the objective, inclusion criteria, study subjects, setting, standard criteria used for measurement of the condition, exposure measurement in validity and reliability strategies to address confounding factors, outcome measurement, and appropriate statistical analysis (Additional file 4). Sensitivity analysis will be conducted to include eligible quality studies in the final systematic review and meta-analysis by investigating the effect size estimates of studies. The quality of the full articles will be assessed by two authors (EW and SB). Any disagreement among reviewers will be resolved by the third author (ZH).

### Data extraction and management

A data extraction template will be constructed in Microsoft Excel (2016) to collect data for the elements of data extraction for full-text eligible manuscripts that will be included in the final systematic review and meta-analysis. The data extraction elements included author name, year of publication, study area, study design, sample size, prevalence or proportion, odds ratio, lower confidence interval, and upper confidence interval. Moreover, new variables will be created to use the elements for analysis using log transformation and standard error on an Excel sheet or STATA 14 after importing the data. Piloting of the data extraction will be carried out before the beginning of the authentic data extraction by all authors. All necessary data will be extracted using the prepiloted Excel data extraction tool. The two authors (EW and SB) will independently extract the data. Any discrepancies will be discussed with a third author (ZH) to reach an agreement. Authors will contact the authors of the studies in case of missing data or incomplete reports.

### Data synthesis and analysis

The extracted data will be imported to STATA version 14 for analysis. The data will be presented using a narrative synthesis of the included studies, and the results will be presented using tables and figures. Square root transformation of data will be performed using the Freeman–Tukey variant of the arcsine to avoid variance variability [32].

The pooled prevalence estimate of male involvement in family planning use in Ethiopia will be performed using a random-effects model [33]. In the random-effects model, we will assume that the true effect size varies from one study to the next and that studies in our analysis will represent a random sample of effect sizes that could have been observed. The summary effect will be our estimate of the mean of these effects. A forest plot will be used to present the pooled prevalence and its determinants of male involvement in family planning use at a statistical significance level of a *p* value of less than 0.05 [34]. Heterogeneity across studies will be assessed using Cochran’s *Q* [35] and *I*^2^ statistics [36]. *I*^2^ values of 25%, 50%, and 75% were representative of low, moderate, and considerable heterogeneity, respectively. Subgroup analysis and meta-regression will be performed based on region and residence (urban and rural) to identify the sources of heterogeneity. Moreover, sensitivity analysis will be performed to investigate the effect size estimates of studies. Publication bias will be checked using visual inspection on the funnel plot [34]. An asymmetry of the funnel plot indicates publication bias. Moreover, Egger’s and Begg’s tests [37] will be conducted to check the potential publication bias, and a *p* value of < 0.05 will be used to declare the statistical significance of publication bias.

## Discussion

This systematic review and meta-analysis protocol aims to synthesize the pooled prevalence of male involvement in family planning use and its determinants in Ethiopia. After the 1994 International Conference on Population and Development (ICPD) and the 1995 UN World Conference on Women, attention to male involvement has improved special efforts to emphasize men’s joint responsibility and promote their active participation in reproductive health serves [1, 11].

Studies have shown that family planning has many benefits, including  reducing maternal, child, and infant mortality; protecting unplanned pregnancy; and improving sustainable socioeconomic development. Family planning could avert up to 42% of maternal mortality [38]. Despite these benefits, contraceptive use is still low, and the unmet need for family planning is high in developing countries, including Ethiopia [4, 7, 9, 10].

Studies in developing countries have examined the role of male involvement in family planning, and male participation improves women’s uptake of family planning methods, increases spousal coordination, supports the success of family planning programs, and provides rights to their partners in reproductive health services [9, 13, 17, 18].

Currently, there are no synthesis research findings on the pooled prevalence of male involvement in family planning use and its determinants in Ethiopia. Therefore, this systematic review and meta-analysis protocol will help the development of appropriate strategies that will have an impact on male involvement in family planning use.

This study protocol may have the following limitations. Heterogeneity may exist between studies due to differences in study designs, settings, sample size, and publication biases. Only articles published in the English language will be considered. Moreover, only observational study designs will be included. Studies conducted in hospital or health care settings will not be representative of the general population.

## Supplementary Information

**Additional file 1.** PRISMA-P 2015 checklist.

**Additional file 2.** Diagrammatic presentation of the studies selection process for systematic review.

**Additional file 3.** Draft of search strategy to be used using PubMed electronic database.

**Additional file 4.** JBI critical appraisals for observational studies as shown in the link below https://jbi.global/critical-appraisal-tools.

## Data Availability

Additional files for the review protocol were submitted as supplementary materials.

## Abbreviations

FP: Family planning
ICPD: International Conference on Population and Development
JBI-MAStARI: Joanna Briggs Institute Meta-Analysis of Statistics Assessment and Review Instrument
PRISMA: Preferred Reporting Items for Systematic Review and Meta-analyses
SDGs: Sustainable development goals
TFR: Total fertility rate
