# Supplementary material for: Male involvement in family planning use and its determinants in Ethiopia: a systematic review and meta-analysis protocol
Source: Syst Rev. 2022 Feb 1;11:19. doi: 10.1186/s13643-022-01891-x (PMC8805394; doi:10.1186/s13643-022-01891-x)
Supplement: Supplementary file 2 — Additional file 2. Diagrammatic presentation of the studies selection process for systematic review. [file 13643_2022_1891_MOESM2_ESM.docx]

**Additional file 2: Diagrammatic presentation of the studies selection process for systematic review.**

| Studies collected using other relevant sources (email request, thesis and USB) (n)  Potentially relevant studies obtain through database search (n)  Duplicated Studies removed (n)  Number of studies after removal of duplications (n)  Papers excluded based on reviewing the title and abstract sections (n)  Studies included based on Title and abstract (n)  Studies eligible for the whole body review for eligibility to be included to the final review (n)  Exclude from the review after assessing the full text of the paper. The papers did not clearly reported prevalence and associated factors (n)  Studies included in the systematic review (n)  Studies included for meta-analysis (n) |
| --- |
