# Supplementary material for: Male involvement in family planning use and its determinants in Ethiopia: a systematic review and meta-analysis protocol
Source: Syst Rev. 2022 Feb 1;11:19. doi: 10.1186/s13643-022-01891-x (PMC8805394; doi:10.1186/s13643-022-01891-x)
Supplement: Supplementary file 3 — Additional file 3. Draft of search strategy to be used using PubMed electronic database. [file 13643_2022_1891_MOESM3_ESM.docx]

**Additional file 3: Draft of search strategy to be used using PubMed electronic database**

| **Search terms** | **Search terms** | **Results** |
| --- | --- | --- |
| Term #1 | (((((((((((((((((("Health Services Accessibility*"[Title/Abstract] AND ((journalarticle[Filter] OR observationalstudy[Filter]) AND (fft[Filter]) AND (humans[Filter]) AND (english[Filter]))) OR ("permanent contraceptive*"[Title/Abstract] AND ((journalarticle[Filter] OR observationalstudy[Filter]) AND (fft[Filter]) AND (humans[Filter]) AND (english[Filter])))) OR ("long-acting contraceptive*"[Title/Abstract] AND ((journalarticle[Filter] OR observationalstudy[Filter]) AND (fft[Filter]) AND (humans[Filter]) AND (english[Filter])))) OR ("Family Planning Services*"[Title/Abstract] AND ((journalarticle[Filter] OR observationalstudy[Filter]) AND (fft[Filter]) AND (humans[Filter]) AND (english[Filter])))) OR ("Contraception Behavior*"[Title/Abstract] AND ((journalarticle[Filter] OR observationalstudy[Filter]) AND (fft[Filter]) AND (humans[Filter]) AND (english[Filter])))) OR ("Family Planning methods*"[Title/Abstract] AND ((journalarticle[Filter] OR observationalstudy[Filter]) AND (fft[Filter]) AND (humans[Filter]) AND (english[Filter])))) OR ("Family Planning*"[Title/Abstract] AND ((journalarticle[Filter] OR observationalstudy[Filter]) AND (fft[Filter]) AND (humans[Filter]) AND (english[Filter])))) OR ("Contraception methods*"[Title/Abstract] AND ((journalarticle[Filter] OR observationalstudy[Filter]) AND (fft[Filter]) AND (humans[Filter]) AND (english[Filter])))) OR (Contraceptive*[Title/Abstract] AND ((journalarticle[Filter] OR observationalstudy[Filter]) AND (fft[Filter]) AND (humans[Filter]) AND (english[Filter])))) OR (Attitudes*[Title/Abstract] AND ((journalarticle[Filter] OR observationalstudy[Filter]) AND (fft[Filter]) AND (humans[Filter]) AND (english[Filter])))) OR ("Health Knowledge*"[Title/Abstract] AND ((journalarticle[Filter] OR observationalstudy[Filter]) AND (fft[Filter]) AND (humans[Filter]) AND (english[Filter])))) OR (Intention*[Title/Abstract] AND ((journalarticle[Filter] OR observationalstudy[Filter]) AND (fft[Filter]) AND (humans[Filter]) AND (english[Filter])))) OR (Practice*[Title/Abstract] AND ((journalarticle[Filter] OR observationalstudy[Filter]) AND (fft[Filter]) AND (humans[Filter]) AND (english[Filter])))) OR ("family Relations*"[Title/Abstract] AND ((journalarticle[Filter] OR observationalstudy[Filter]) AND (fft[Filter]) AND (humans[Filter]) AND (english[Filter])))) OR (Communication*[Title/Abstract] AND ((journalarticle[Filter] OR observationalstudy[Filter]) AND (fft[Filter]) AND (humans[Filter]) AND (english[Filter])))) OR ("Decision Making*"[Title/Abstract] AND ((journalarticle[Filter] OR observationalstudy[Filter]) AND (fft[Filter]) AND (humans[Filter]) AND (english[Filter])))) OR ("Men involvement*"[Title/Abstract] AND ((journalarticle[Filter] OR observationalstudy[Filter]) AND (fft[Filter]) AND (humans[Filter]) AND (english[Filter])))) OR ("male involvement*"[Title/Abstract] AND ((journalarticle[Filter] OR observationalstudy[Filter]) AND (fft[Filter]) AND (humans[Filter]) AND (english[Filter])))) OR ("Husbands’ involvement*"[Title/Abstract] AND ((journalarticle[Filter] OR observationalstudy[Filter]) AND (fft[Filter]) AND (humans[Filter]) AND (english[Filter]))) |  |
| Term #2 | ((((((((determinates*[Title/Abstract] AND ((journalarticle[Filter] OR observationalstudy[Filter]) AND (fft[Filter]) AND (humans[Filter]) AND (english[Filter]))) OR (predictors*[Title/Abstract] AND ((journalarticle[Filter] OR observationalstudy[Filter]) AND (fft[Filter]) AND (humans[Filter]) AND (english[Filter])))) OR ("associated factors*"[Title/Abstract] AND ((journalarticle[Filter] OR observationalstudy[Filter]) AND (fft[Filter]) AND (humans[Filter]) AND (english[Filter])))) OR ("Educational Status*"[Title/Abstract] AND ((journalarticle[Filter] OR observationalstudy[Filter]) AND (fft[Filter]) AND (humans[Filter]) AND (english[Filter])))) OR ("Socioeconomic Factors*"[Title/Abstract] AND ((journalarticle[Filter] OR observationalstudy[Filter]) AND (fft[Filter]) AND (humans[Filter]) AND (english[Filter])))) OR ("Age Factors*"[Title/Abstract] AND ((journalarticle[Filter] OR observationalstudy[Filter]) AND (fft[Filter]) AND (humans[Filter]) AND (english[Filter])))) OR ("gender identity*"[Title/Abstract] AND ((journalarticle[Filter] OR observationalstudy[Filter]) AND (fft[Filter]) AND (humans[Filter]) AND (english[Filter])))) OR (Education*[Title/Abstract] AND ((journalarticle[Filter] OR observationalstudy[Filter]) AND (fft[Filter]) AND (humans[Filter]) AND (english[Filter])))) OR ((Marriage*[Title/Abstract]) AND (Sex[Title/Abstract]) AND ((journalarticle[Filter] OR observationalstudy[Filter]) AND (fft[Filter]) AND (humans[Filter]) AND (english[Filter]))) |  |
| Term #3 | (((((((((Spouses*[Title/Abstract] AND ((journalarticle[Filter] OR observationalstudy[Filter]) AND (fft[Filter]) AND (humans[Filter]) AND (english[Filter]))) OR (men*[Title/Abstract] AND ((journalarticle[Filter] OR observationalstudy[Filter]) AND (fft[Filter]) AND (humans[Filter]) AND (english[Filter])))) OR (Male*[Title/Abstract] AND ((journalarticle[Filter] OR observationalstudy[Filter]) AND (fft[Filter]) AND (humans[Filter]) AND (english[Filter])))) OR ("Young Adult*"[Title/Abstract] AND ((journalarticle[Filter] OR observationalstudy[Filter]) AND (fft[Filter]) AND (humans[Filter]) AND (english[Filter])))) OR (Adolescent*[Title/Abstract] AND ((journalarticle[Filter] OR observationalstudy[Filter]) AND (fft[Filter]) AND (humans[Filter]) AND (english[Filter])))) OR ("middle-aged*"[Title/Abstract] AND ((journalarticle[Filter] OR observationalstudy[Filter]) AND (fft[Filter]) AND (humans[Filter]) AND (english[Filter])))) OR (Female*[Title/Abstract] AND ((journalarticle[Filter] OR observationalstudy[Filter]) AND (fft[Filter]) AND (humans[Filter]) AND (english[Filter])))) OR ("Adult Aged*"[Title/Abstract] AND ((journalarticle[Filter] OR observationalstudy[Filter]) AND (fft[Filter]) AND (humans[Filter]) AND (english[Filter])))) OR (Adult*[Title/Abstract] AND ((journalarticle[Filter] OR observationalstudy[Filter]) AND (fft[Filter]) AND (humans[Filter]) AND (english[Filter])))) OR (Humans*[Title/Abstract] AND ((journalarticle[Filter] OR observationalstudy[Filter]) AND (fft[Filter]) AND (humans[Filter]) AND (english[Filter]))) |  |
| Term#4 | Ethiopia*[Title/Abstract] AND ((journalarticle[Filter] OR observationalstudy[Filter]) AND (fft[Filter]) AND (humans[Filter]) AND (english[Filter])) |  |
| Term #5 | #1 AND #2 AND #3 AND #4=  (((Ethiopia*[Title/Abstract] AND ((journalarticle[Filter] OR observationalstudy[Filter]) AND (fft[Filter]) AND (humans[Filter]) AND (english[Filter]))) AND ((((((((((Spouses*[Title/Abstract] AND ((journalarticle[Filter] OR observationalstudy[Filter]) AND (fft[Filter]) AND (humans[Filter]) AND (english[Filter]))) OR (men*[Title/Abstract] AND ((journalarticle[Filter] OR observationalstudy[Filter]) AND (fft[Filter]) AND (humans[Filter]) AND (english[Filter])))) OR (Male*[Title/Abstract] AND ((journalarticle[Filter] OR observationalstudy[Filter]) AND (fft[Filter]) AND (humans[Filter]) AND (english[Filter])))) OR ("Young Adult*"[Title/Abstract] AND ((journalarticle[Filter] OR observationalstudy[Filter]) AND (fft[Filter]) AND (humans[Filter]) AND (english[Filter])))) OR (Adolescent*[Title/Abstract] AND ((journalarticle[Filter] OR observationalstudy[Filter]) AND (fft[Filter]) AND (humans[Filter]) AND (english[Filter])))) OR ("middle-aged*"[Title/Abstract] AND ((journalarticle[Filter] OR observationalstudy[Filter]) AND (fft[Filter]) AND (humans[Filter]) AND (english[Filter])))) OR (Female*[Title/Abstract] AND ((journalarticle[Filter] OR observationalstudy[Filter]) AND (fft[Filter]) AND (humans[Filter]) AND (english[Filter])))) OR ("Adult Aged*"[Title/Abstract] AND ((journalarticle[Filter] OR observationalstudy[Filter]) AND (fft[Filter]) AND (humans[Filter]) AND (english[Filter])))) OR (Adult*[Title/Abstract] AND ((journalarticle[Filter] OR observationalstudy[Filter]) AND (fft[Filter]) AND (humans[Filter]) AND (english[Filter])))) OR (Humans*[Title/Abstract] AND ((journalarticle[Filter] OR observationalstudy[Filter]) AND (fft[Filter]) AND (humans[Filter]) AND (english[Filter]))) AND ((journalarticle[Filter] OR observationalstudy[Filter]) AND (fft[Filter]) AND (humans[Filter]) AND (english[Filter])))) AND (((((((((determinates*[Title/Abstract] AND ((journalarticle[Filter] OR observationalstudy[Filter]) AND (fft[Filter]) AND (humans[Filter]) AND (english[Filter]))) OR (predictors*[Title/Abstract] AND ((journalarticle[Filter] OR observationalstudy[Filter]) AND (fft[Filter]) AND (humans[Filter]) AND (english[Filter])))) OR ("associated factors*"[Title/Abstract] AND ((journalarticle[Filter] OR observationalstudy[Filter]) AND (fft[Filter]) AND (humans[Filter]) AND (english[Filter])))) OR ("Educational Status*"[Title/Abstract] AND ((journalarticle[Filter] OR observationalstudy[Filter]) AND (fft[Filter]) AND (humans[Filter]) AND (english[Filter])))) OR ("Socioeconomic Factors*"[Title/Abstract] AND ((journalarticle[Filter] OR observationalstudy[Filter]) AND (fft[Filter]) AND (humans[Filter]) AND (english[Filter])))) OR ("Age Factors*"[Title/Abstract] AND ((journalarticle[Filter] OR observationalstudy[Filter]) AND (fft[Filter]) AND (humans[Filter]) AND (english[Filter])))) OR ("gender identity*"[Title/Abstract] AND ((journalarticle[Filter] OR observationalstudy[Filter]) AND (fft[Filter]) AND (humans[Filter]) AND (english[Filter])))) OR (Education*[Title/Abstract] AND ((journalarticle[Filter] OR observationalstudy[Filter]) AND (fft[Filter]) AND (humans[Filter]) AND (english[Filter])))) OR ((Marriage*[Title/Abstract]) AND (Sex[Title/Abstract]) AND ((journalarticle[Filter] OR observationalstudy[Filter]) AND (fft[Filter]) AND (humans[Filter]) AND (english[Filter]))) AND ((journalarticle[Filter] OR observationalstudy[Filter]) AND (fft[Filter]) AND (humans[Filter]) AND (english[Filter])))) AND ((((((((((((((((((("Health Services Accessibility*"[Title/Abstract] AND ((journalarticle[Filter] OR observationalstudy[Filter]) AND (fft[Filter]) AND (humans[Filter]) AND (english[Filter]))) OR ("permanent contraceptive*"[Title/Abstract] AND ((journalarticle[Filter] OR observationalstudy[Filter]) AND (fft[Filter]) AND (humans[Filter]) AND (english[Filter])))) OR ("long-acting contraceptive*"[Title/Abstract] AND ((journalarticle[Filter] OR observationalstudy[Filter]) AND (fft[Filter]) AND (humans[Filter]) AND (english[Filter])))) OR ("Family Planning Services*"[Title/Abstract] AND ((journalarticle[Filter] OR observationalstudy[Filter]) AND (fft[Filter]) AND (humans[Filter]) AND (english[Filter])))) OR ("Contraception Behavior*"[Title/Abstract] AND ((journalarticle[Filter] OR observationalstudy[Filter]) AND (fft[Filter]) AND (humans[Filter]) AND (english[Filter])))) OR ("Family Planning methods*"[Title/Abstract] AND ((journalarticle[Filter] OR observationalstudy[Filter]) AND (fft[Filter]) AND (humans[Filter]) AND (english[Filter])))) OR ("Family Planning*"[Title/Abstract] AND ((journalarticle[Filter] OR observationalstudy[Filter]) AND (fft[Filter]) AND (humans[Filter]) AND (english[Filter])))) OR ("Contraception methods*"[Title/Abstract] AND ((journalarticle[Filter] OR observationalstudy[Filter]) AND (fft[Filter]) AND (humans[Filter]) AND (english[Filter])))) OR (Contraceptive*[Title/Abstract] AND ((journalarticle[Filter] OR observationalstudy[Filter]) AND (fft[Filter]) AND (humans[Filter]) AND (english[Filter])))) OR (Attitudes*[Title/Abstract] AND ((journalarticle[Filter] OR observationalstudy[Filter]) AND (fft[Filter]) AND (humans[Filter]) AND (english[Filter])))) OR ("Health Knowledge*"[Title/Abstract] AND ((journalarticle[Filter] OR observationalstudy[Filter]) AND (fft[Filter]) AND (humans[Filter]) AND (english[Filter])))) OR (Intention*[Title/Abstract] AND ((journalarticle[Filter] OR observationalstudy[Filter]) AND (fft[Filter]) AND (humans[Filter]) AND (english[Filter])))) OR (Practice*[Title/Abstract] AND ((journalarticle[Filter] OR observationalstudy[Filter]) AND (fft[Filter]) AND (humans[Filter]) AND (english[Filter])))) OR ("family Relations*"[Title/Abstract] AND ((journalarticle[Filter] OR observationalstudy[Filter]) AND (fft[Filter]) AND (humans[Filter]) AND (english[Filter])))) OR (Communication*[Title/Abstract] AND ((journalarticle[Filter] OR observationalstudy[Filter]) AND (fft[Filter]) AND (humans[Filter]) AND (english[Filter])))) OR ("Decision Making*"[Title/Abstract] AND ((journalarticle[Filter] OR observationalstudy[Filter]) AND (fft[Filter]) AND (humans[Filter]) AND (english[Filter])))) OR ("Men involvement*"[Title/Abstract] AND ((journalarticle[Filter] OR observationalstudy[Filter]) AND (fft[Filter]) AND (humans[Filter]) AND (english[Filter])))) OR ("male involvement*"[Title/Abstract] AND ((journalarticle[Filter] OR observationalstudy[Filter]) AND (fft[Filter]) AND (humans[Filter]) AND (english[Filter])))) OR ("Husbands’ involvement*"[Title/Abstract] AND ((journalarticle[Filter] OR observationalstudy[Filter]) AND (fft[Filter]) AND (humans[Filter]) AND (english[Filter]))) AND ((journalarticle[Filter] OR observationalstudy[Filter]) AND (fft[Filter]) AND (humans[Filter]) AND (english[Filter]))) |  |
